# Nutritional Status and Related Factors in Patients with Gastric Cancer after Gastrectomy: A Cross-Sectional Study

**DOI:** 10.3390/nu14132634

**Published:** 2022-06-25

**Authors:** Hui-Mei Wang, Tsae-Jyy Wang, Ching-Shui Huang, Shu-Yuan Liang, Chia-Hui Yu, Ting-Ru Lin, Kuo-Feng Wu

**Affiliations:** 1 Department of Nursing, Cathay General Hospital, Taipei 106, Taiwan; cgh259980@cgh.org.tw; 2 School of Nursing, National Taipei University of Nursing and Health Sciences, Taipei 112, Taiwan; shuyuan@ntunhs.edu.tw (S.-Y.L.); r0004467@gmail.com (T.-R.L.); 3 Consultant for Medical Affairs, Department of Surgery, Cathay General Hospital, Taipei 106, Taiwan; cshuang@cgh.org.tw; 4 Department of Obstetrics and Gynecology, Mackay Memorial Hospital, Taipei 104, Taiwan; y6635158.6627@mmh.org.tw; 5 Department of Nurse-Midwifery and Women Health, National Taipei University of Nursing and Health Science, Taipei 112, Taiwan; kuofeng@nutunhs.edu.tw

**Keywords:** diet preparation, nutritional status, gastric cancer, gastrectomy, symptom

## Abstract

Patients after gastrectomy for gastric cancer are at risk of malnutrition, and poor nutritional status negatively affects patients’ clinical outcomes. Knowledge of the factors influencing patients’ nutritional status can inform interventions for improving patients’ nutrition. A cross-sectional study was conducted to describe nutritional status and related factors in gastric cancer patients after gastrectomy. A convenience sample of gastric cancer patients with gastrectomy was recruited from general surgery or oncology clinics of a medical center in northern Taiwan. Data were collected with self-reported questionnaires, including the Functional Assessment Cancer Therapy—Gastric Module version 4, the Concerns in Meal Preparation scale, the Center for Epidemiologic Studies Depression Scale, and the Mini Nutrition Assessment. One hundred and one gastric cancer patients participated in the study. There were 81 cases of subtotal gastrectomy and 20 cases of total gastrectomy. Most patients (52.5%) were malnourished or at risk. Linear regression showed that symptom severity (*β* = −0.43), employment status (*β* = 0.19), and difficulty in diet preparation (*β* = −0.21) were significant predictors of nutritional status. Together, these three variables explained 35.8% of the variance in patient nutritional status (*F* = 20.3, *p* < 0.001). More than 50% of our participants were malnourished or at risk for malnutrition, indicating a need for continued monitoring and support after discharge from hospitals. Special attention should be given to patients with severe symptoms, unemployment, and difficulties in diet preparation.

## 1. Introduction

Gastric cancer is the fifth most commonly diagnosed cancer worldwide, with approximately 1 million diagnosed in 2018 [1]. It is also responsible for the third-largest number of deaths. About 0.78 million deaths were counted in 2018 [1]. In Taiwan, gastric cancer is the ninth most commonly diagnosed cancer, with an incidence rate of 9.5 per 100 thousand people in 2016 [2]. It is responsible for the seventh-largest number of deaths, with a mortality rate of 9.7 per 100 thousand people in 2018 [3]. 

Total or subtotal gastrectomy is the primary therapeutic approach for Gastric cancer [4]. These surgical procedures can disrupt patients’ gastrointestinal anatomy and physiological function, affecting nutrient absorption and leading to gastrointestinal symptoms [5,6]. After surgery, patients often experience a lack of appetite, feeling full after eating a small amount of food, or gastroesophageal reflux. They may even experience symptoms such as nausea, vomiting, bloating, abdominal pain, and diarrhea or suffer dysphagia [5]. Severe symptoms can cause further psychological distress [7] and negatively impact the patient’s food intake [8,9]. Emotional distress, symptom disturbance, and cancer-related inflammation can increase protein energy-wasting, which leads to reduced body weight [10]. After gastrectomy, most patients have insufficient protein and calorie intake and continue to experience weight loss for up to six months [11]. One year after surgery, 21.4% of patients (*n* = 1905) still suffer from malnutrition [12]. Weight loss and malnutrition negatively affect patients’ outcomes [13,14]. Knowledge of the factors influencing patients’ nutritional status after gastric cancer surgery can inform interventions for improving patients’ nutrition and outcomes.

Patients who underwent gastrectomy may face difficulties preparing their diets after returning home from the hospital [15]. Problems in diet preparation can negatively impact a patient’s nutritional status. A study of patients with breast cancer showed that it was difficult to change their dietary habits while sharing a life with other family members who had regular diets. It was sometimes tiring enough for those living alone to get out of the house and have takeaway food, let alone prepare nutritious food [16]. These previous studies showed that diet preparation could be a challenge for patients with cancer. However, to the best of our knowledge, there is no relevant study on difficulties with diet preparation in patients with gastric cancer after surgery and the influence of these difficulties on their nutritional status.

In summary, due to increased metabolic demand, insufficient nutrient intake, or nutrient loss, patients who underwent gastrectomy for gastric cancer may suffer from malnutrition and weight loss after returning home [17]. Low nutritional status harms an individual clinical outcome [18]. Knowledge regarding factors influencing patients’ nutritional status can inform the development of appropriate measures to improve patients’ nutrition quality. Previous studies have shown that gastrointestinal symptoms [8], depression [7], and difficulties with diet preparation [19] might affect the nutrient intake of patients. However, most of these studies were conducted in western countries on patients with other cancers. Few explored difficulties with diet preparation of patients with gastric cancer after being discharged and returning home. Therefore, this study aimed to investigate the nutritional status of patients who underwent gastrectomy and the factors associated with this status, including demographics, disease profiles, gastrointestinal symptoms, depression, and difficulties in preparing a post-gastrectomy diet. 

## 2. Materials and Methods

### 2.1. Study Design

The study uses a cross-sectional study design. The study was launched after acquiring approval from the hospital’s ethics committee. 

### 2.2. Samples and Location

A convenience sample of gastric cancer patients was recruited from a medical center’s general surgery or oncology clinics in Taiwan. Potential participants were identified through an outpatient information system. A nursing researcher recruited patients who met the following eligibility criteria from the clinics. The inclusion criteria are: (1) aged 20 or older, (2) diagnosed with gastric cancer, (3) having undergone total or subtotal gastrectomy, (4) time since surgery between three months and two years, (5) able to communicate in Taiwanese or Mandarin, and (6) able to eat orally. The exclusion criteria were: (1) needing long-term gastroesophageal feeding, (2) suffering severe mental disorders, (3) currently receiving chemotherapy or radiation therapy, and (4) with distant metastasis. 

### 2.3. Data Collection and Instruments

Every participant signed informed consent before taking part in the study. Data were collected using self-report questionnaires. The questionnaires were administered to the participants in a quiet room in the outpatient clinic after their clinical visits. One of the researchers read each question to the participant, and the participant wrote the responses on their own. The researcher filled out their verbal responses if a participant could not write. The researcher collected data on disease- and therapy-related characteristics from participants’ medical records. 

The study questionnaires included demographics and disease characteristics, Gastric Cancer Subscale of the Functional Assessment Cancer Therapy—Gastric Module version 4, Concerns in Diet Preparation scale, Center for Epidemiologic Studies Depression Scale (CES-D), and Mini Nutrition Assessment (MNA). The demographic data collected include gender, age, education level, marital status, financial situation, living conditions, and diet preparation. The disease- and therapy-related characteristics collected include the histological type of cancer, cancer staging, surgical procedure, and adjuvant therapies. 

The Gastric Cancer Subscale of the Functional Assessment Cancer Therapy—Gastric (FACT-Ga) (version 4) [20] was used to assess the participants’ symptoms and adverse effects associated with gastric cancer treatment in the past seven days. The scale has 19 items rated on a 5-point Likert-type scale (0, not at all; 4, very much). Summing up item scores yields the scale’s score, with a possible range of 0 to 76. A higher score signified a more severe case of gastric cancer symptoms. The scale provides valid and reliable measurement of symptom and treatment adverse effects in gastric cancer patients [21,22]. The Cronbach’s α of the scale is 0.88 in this sample.

The Concerns in Diet Preparation scale [23] was used to measure the difficulties encountered during diet preparation. The scale was developed in Chinese and had 13 items rated on a 1 (never) to 5 (always) Likert-type scale (Table A1). The sum of item scores represents the scale score with a possible range of 13 to 65. The higher the score, the greater the difficulties encountered by the participants. The CVI of the scale was 0.97. The Cronbach’s α of the scale was 0.83 in a previous study of patients with liver cancer (*n* = 88) [23]. Cronbach’s α of the scale is 0.87 in this study.

The Chinese version of the Center for Epidemiologic Studies Depression Scale (CES-D) [24] was used to assess the participants’ depression severity. The scale includes 20 items rated on a 4-point Likert-type scale (0 to 3). Reverse scoring the negative items and summing up item scores gives the scale’s score, which could range from 0 to 60. A score of 16 can be used as the cutoff on the CES-D for depression [24]. The scale has been widely used in clinical populations with good reliability and validity [25,26]. The Cronbach’s αof the scale is 0.86 in this study.

The Chinese version of the Mini Nutrition Assessment (MNA) [27] assessed the participants’ nutritional status. The 18-item scale includes anthropometric measurements, general assessment (lifestyle, medication use, and mobility), dietary assessment, and self-perceived health and nutritional status. Each item adopted a different scoring method, including 2-point (0 to 1), 3-point (0 to 2), and 4-point (0 to 3) scoring methods. The sum of item scores represents the scale score with a possible range of 0 to 30. A score between 17 to 23.5 indicates a potential risk of malnutrition. A score lower than 17 indicates malnutrition. A score equal to or higher than 24 suggests an excellent nutritional status [28,29]. The scale has been widely used in cancer populations and has demonstrated good reliability and validity [29,30]. The Cronbach’s α of the scale is 0.71 in this current sample.

### 2.4. Sample Size Estimation

The required sample size was estimated using G-power software 3.1. The estimation was based on a multivariate regression model with ten predictors, a medium (f^2^ = 0.15) effect size, and a significance level of 0.05. A sample of 118 is required to have 80% statistical power. 

### 2.5. Data Analysis

SPSS version 22.0 was used to analyze the data. The study variables were described using the frequency, percentage, mean, and standard deviation. A t-test and ANOVA were used to examine nutritional status differences among participants with different demographics and disease profiles. Pearson’s product-moment correlation coefficient was used to analyze the correlations between gastrointestinal symptom severity, depression severity, difficulties with diet preparation, and nutritional status after gastrectomy. Stepwise multiple linear regression analysis was used to identify significant predictors of participants’ nutritional status after gastrectomy. Categorical variables were dummy coded before analysis. All the study variables were entered into the regression model as the independent variables. The selection of predictors was performed through the Forward (Criterion: Probability-of-F-to-enter ≤ 0.050) selection procedure. Standardized residual plots and collinearity statistics were used to examine normality, equal variance, and independence assumptions. All these assumptions were not violated.

## 3. Results

One of the nursing researchers reviewed 217 medical records of patients with gastric cancer from the inpatient information system and contacted 146 potentially eligible patients at the outpatient clinics. Forty-two were excluded due to not meeting the study eligibility criteria, and three refused to participate. A total of 101 post-gastrectomy patients participated in the study. There were no missing data in this study.

### 3.1. Participants’ Characteristics

The average age of the participants was 66.5 (SD = 14.0; Table 1). Majority of them were male (*n* = 53), married (*n* = 92), with a primary or lower level of education (*n* = 40), and unemployed (*n* = 81). Most participants lived together with other family members or relatives (*n* = 96). The average duration after gastrectomy surgery was 10.9 months (SD = 7.6). Their cancer histologic types include adenocarcinoma (*n* = 74), Singent-cell carcinoma (*n* = 19), and gastrointestinal stromal tumors (GIST, *n* = 8). Thirty-five participants had stage 0 to I cancer, 26 had stage II cancer, and 40 had stage III cancer according to the TNM classification system of malignant tumors [31].

The surgical procedures included subtotal gastrectomy with B-II anastomosis (*n* = 65), subtotal gastrectomy with B-I anastomosis (*n* = 16), and total gastrectomy (*n* = 20). The adjuvant therapy received included oral chemotherapy (*n* = 27), intravenous chemotherapy (*n* = 18), and adjuvant concurrent chemoradiation therapy (CCRT, *n* = 6). 

### 3.2. Symptom Severity, Diet Preparation Difficulties, Depression Severity, and Nutritional Status

The participants scored 16.7 (SD = 11.6) on average for the Gastric Cancer Subscale of the FACT-Ga, ranging from 1 to 59. Among all the symptoms, participants were most concerned about not being able to eat their favorite foods, avoiding eating out due to illness, and being troubled by gas (flatulence; Table 2).

The participants had an average score of 22.3 (SD = 8.1) on the Concerns in Diet Preparation scale, ranging from 13 to 45. The participants scored 9.6 points (SD = 7.6) on average on the CESD scale, ranging from 0 to 29 (Table 1). Using a score of 16 as the cutoff point, 21 participants had clinical depression. The participants scored 22.8 points (SD = 4.0) on average on the MNA scale, ranging from 8.5 to 28.5 (Table 1). Among them, 48 participants scored equal to or greater than 24, indicating well-nourished; 44 participants scored between 17 and 23.5, indicating the risk of malnutrition; nine participants scored less than 17 points, suggesting malnourished.

### 3.3. Factors Associated with Nutritional Status

Results of bivariate analysis show that nutritional statuses were significant different among participants with different gender (*t* = 1.99, *p* = 0.049), working status (*t* = −4.07, *p* < 0.001), stage of cancer (*F* = 5.4, *p* = 0.006), and surgical procedures (*F* = 3.7, *p* = 0.029). Female participants had better nutritional status than male participants (23.6 vs. 22.0). Participants with a job had better nutritional status than those without (25.0 vs. 22.2). Scheffe’s post hoc tests showed that participants with stage 0 or stage I cancer had better nutritional status than those with stage II (24.5 vs. 21.7) or III cancer (24.5 vs. 22.1). Scheffe’s post hoc tests showed that participants who had undergone Subtotal B-I partial gastrectomy (with the stomach reconnected to the duodenum) had better nutritional status than those who had undergone Subtotal B-II partial gastrectomy (with the stomach reconnected to the jejunum; 23.9 vs. 23.2) as well as those having undergone total gastrectomy (23.9 vs. 20.8). There were no statistically significant differences in participants’ nutritional status with differences in the other demographics and disease profiles, including education level, marital status, living arrangement, histological type of cancer, and adjuvant therapies (Table 3).

**Figure 1:**
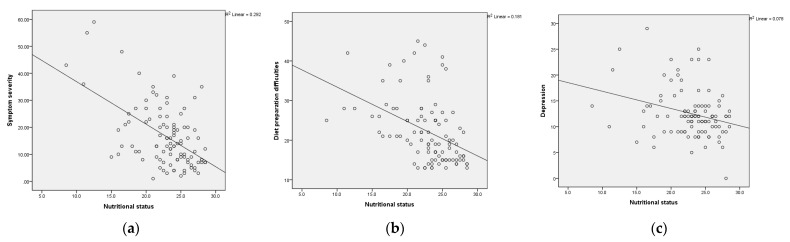
Scatterplot of (**a**) nutritional status with symptom severity; (**b**) nutritional status with diet preparation difficulties; (**c**) nutritional status with depression severity.

Result of Pearson’s correlation analysis showed that nutritional status negatively correlated with symptom severity (*r* = −0.54, *p* < 0.01), depression severity (*r* = −0.40, *p* < 0.001), and diet preparation difficulties (*r* = −0.43, *p* < 0.01; Table 4). Participants with more severe symptoms, greater depression, and more difficulty preparing diets had worse nutritional status (Figure 1). Age and disease duration were not significantly associated with nutritional status (Table 4).

### 3.4. Predictive Factors of Nutritional Status

Stepwise multiple linear regression analysis was used to identify important predictors of nutritional status. Independent variables entered into the regression model included age, gender, marital status, employment status, living arrangement, education level, disease duration, disease staging, surgical procedure, symptom severity, depression severity, and difficulties in diet preparation. Due to weak associations found among symptom severity, depression severity, and concerns in diet preparation in the correlation analysis, the interaction terms between these variables were also entered into the stepwise regression model. Symptom severity was the first variable entering the regression model, explaining 28.4% of the variance in nutritional status. Employment status was the second variable entering the model and could explain 4.4% of the nutritional status variance. Diet preparation difficulty was the last variable entering the model and could explain an extra 3% of the nutritional status variance. The final model showed that symptom severity (*β* = −0.42), employment status (*β* = 0.19), and diet preparation difficulties (*β* = −0.21) were significant predictors for gastric cancer patients’ nutritional status. The three variables together could explain 35.8% of the variability in nutritional status (*F* = 20.3, *p* < 0.001; Table 5).

## 4. Discussion

### 4.1. Gastric Cancer Patients’ Nutritional Status

Low nutritional status is a particular issue among patients who underwent gastrectomy for gastric cancer. We investigated the nutritional status of 101 gastric cancer patients with an average of 10.94 months after gastrectomy. Among them, 47.5% were well-nourished, 43.6% were at risk for malnutrition, and 8.9% were malnourished, according to their MNA scores. The proportions of patients at risk for malnutrition were higher than reported in a previous study. Aaldriks et al. [32] investigated 202 cancer patients receiving chemotherapy, of which 65% were well-nourished, 30% were at risk for malnutrition, and 5% were malnourished. However, Aaldriks et al. [32] enrolled patients with various types of malignancy (breast, colorectal, ovarian cancer, etc.) compared to only gastric cancer patients enrolled in the current study. Our study participants shared similar nutritional status as the patients (*n* = 1905) in Lee et al. [12], who had gastric cancer for five months, and 21.4% of them were found to have mild malnutrition.

### 4.2. Factors Associated with Nutritional Status

Similar to what was reported in a previous study [33], our study results also showed that women had significantly better nutritional status than men. However, the gender difference in nutritional status found in the present study may be partially explained by different disease severity among patients of a different gender. In our study, 21.8% of the male participants had stage III gastric cancer, but only 7.9% of the female participants had stage III cancer. 

We found that participants who stayed employed after surgery had better nutritional status than those who were unemployed. This finding could be because gastric cancer patients with low nutritional status experienced less strength and lack of energy to maintain their jobs [34]. It is also possible that those with better social and economic status had more resources for maintaining nutritional status [35]. 

Not surprised, we found participants with stage 0 and stage I cancer had significantly better nutritional status than those with stage II and stage III cancers. Later stage cancers are more invasive, featuring a higher metabolic demand and more rapid cell reproduction, increasing energy metabolism and low nutritional status. Similar to the finding of Ushimaru et al.’s [36] study, we found that patients with partial gastrectomy had significantly better nutritional status than those with total gastrectomy. Irregular bowel movements and abnormal intestinal hormone regulation resulting from total gastrectomy could partially explain this finding [37].

Symptom severity was negatively associated with nutritional status (*r* = −0.54, *p* < 0.01), suggesting that patients with more severe gastrointestinal symptoms suffer a lower nutritional status. Similar to what was reported in a previous study, discomforts experienced after eating and lack of appetite led to malnutrition in patients after surgery [38]. We found that changing eating habits was one of the patients’ most concerning issues and often prevented them from enjoying meals with their families. Molassiotis et al. [39] also reported that patients felt isolated due to dietary changes after surgery and needed to adjust to new food types. They could not eat what used to be their favorite foods and had a diet different from their family. In addition, Grace et al. [38] reported that gastrointestinal tract symptoms were the most concerning issue for postoperative gastric cancer patients. 

Depression severity was negatively associated with nutritional status (*r* = −0.28, *p* = 0.005), suggesting that more severe depression patients have a more inferior nutritional status. Depression could negatively affect patients’ appetite and influence intake [9]. Previous studies [17,40] also reported that depression experienced by patients with gastric cancer could affect their nutritional status. However, overall, the depressed mood of our study participants was not very severe. The average score for CES-D was 12.6, and only 18.8% of the study participants were found with a depressive condition (CESD > 16). 

We found a negative correlation between diet preparation difficulties and nutritional status (*r* = −0.43, *p* < 0.001), suggesting patients with a higher difficulty level would have more inferior nutritional status. This result is consistent with the findings of Grace et al. [38]. Our participants scored an average of 22.3 (SD = 8.1) on the diet preparation difficulties scale with a possible range of 13 to 65, indicating a medium level of difficulty preparing a diet. They reported the most difficulties in fear of feeling uncomfortable after eating, not knowing what food choices are better for their health, and not knowing how much to eat to gain enough nutrition. The participants also reported avoiding eating certain foods because they believed these would cause the tumor to grow larger. These included raw foods, alcohol, chili pepper, duck meat, and smoked foods. 

Our results support the findings that symptoms and employment status were significant predictors of nutritional status. Gastric cancer patients who were unemployed after surgery had more severe symptoms and more significant difficulties in preparing diet and were at the greatest risk for low nutritional status. Gastrectomy changes a patient’s gastrointestinal structures and physiological functions. Many patients have a poor appetite and experience bloating and fullness after eating. Symptoms and adverse effects of gastric cancer treatment negatively affect a patient’s calorie intake and nutritional status [38]. Patients often need to change their dietary habits and adjust to the food intake restraints posed by their conditions. They are also unsure which foods to choose for adequate nutrients and better health. Difficulties in preparing a diet further compromise one’s nutritional status. 

### 4.3. Study Limitations

Its cross-sectional design and non-probability sampling limit the study. The cross-sectional study design only allows for the determination of associations and cannot make any causal inferences between the variables studied. For example, although this study showed that unemployed participants had worse nutritional status than participants with jobs, it was unclear whether the employment status had affected their nutritional status or whether patients had to resign from their job due to low nutritional status. The cross-sectional nature of the study also precluded assessing the nutritional status changes over time and their impact on recovery. Furthermore, we did not collect data on nutritional status at the time of cancer diagnosis, excluding the possibility of controlling for it as a covariate in regression models. Lastly, this study recruited a convenience sample from a single medical center, and the results cannot be inferred for all gastric cancer patients. Nevertheless, the study results provide important information about factors influencing the nutritional status of gastric cancer patients.

## 5. Conclusions

Gastric cancer patients are at risk for malnutrition after gastrectomy, and their nutritional status should be evaluated closely. More than 50% of our participants were malnourished or at risk for malnutrition, indicating a need for continued monitoring and support after discharge from hospitals. Patients with severe symptoms, unemployment, and more difficulties in preparing diet are at significant risk for malnutrition. Clinicians should pay particular attention to these high-risk groups. Interventions to relieve gastrointestinal symptoms and strategies to reduce diet preparation difficulties are also recommended for improving nutritional status in postoperative gastric cancer patients. These include addressing common concerns and discomforts found in this study: not being able to eat favorite foods, avoiding eating out due to illness, being troubled by gas (bloating), not being able to eat with family or friends, and feeling full or having a heavy stomach. Common challenges in diet preparation to overcome include: the fear of feeling uncomfortable after eating, not knowing what food choices are better for my health, not knowing how much to eat to gain enough nutrition, not knowing which foods I should not eat, and worrying about eating too much nutrition can make tumors bigger and more serious. 

## Figures and Tables

**Table 1:** Participants’ characteristics and descriptive data of study variables (N = 101).

Variables	Mean	SD	Range
Age	66.5	14.0	25–89
Disease duration (month)	10.9	7.6	3–23
Symptom severity	16.7	11.6	1–59
Diet preparation difficulties	22.3	8.1	13–45
Depression severity	9.6	7.6	0–29
Nutritional status	22.8	4.0	8.5–28.5

**Table 2:** Participants’ symptoms and adverse effects associated with gastric cancer treatment (N = 101).

Items	Mean	SD	Range
Unable to eat the foods that I like	1.65	1.22	0–4
Avoid eating out due to illness	1.51	1.35	0–4
Being bothered by gas (flatulence)	1.31	1.34	0–4
Unable to enjoy meals with family or friends	1.20	1.35	0–4
Having stomach problems that worry me	1.05	1.13	0–4
Having fullness or heaviness in the stomach	1.01	1.09	0–4
Having discomfort or pain when eating.	0.94	1.06	0–4
Bothered by a change in eating habits	0.92	1.06	0–4
Feeling tired	0.93	1.11	0–4
Having swelling or cramps in the stomach area	0.88	1.12	0–4
Having discomfort or pain in the stomach area	0.89	1.02	0–4
Bothered by reflux or heartburn	0.72	1.04	0–3
Losing weight	0.40	0.86	0–4
Loss of appetite	0.66	1.09	0–4
Having trouble swallowing food	0.28	0.74	0–4
Interfering with usual activities by digestive problems	0.68	0.95	0–4
Having diarrhea (diarrhoea)	0.49	0.84	0–3
Feeling weak all over.	0.63	1.06	0–4
Difficulty planning for the future because of illness	0.57	0.98	0–4
Total score	16.7	11.6	1–59

**Table 3:** Differences in nutritional status among patients with different demographics and disease profiles (*n* = 101).

Variable			Nutritional Status		
		*n*	Mean (SD)	*t*/F	*p*
Gender				1.99	0.049 *
	Male	53	23.6 (4.2)		
	Female	48	22.0 (3.6)		
Education level				2.12	0.125
	①Elementary and below	40	22.5 (3.6)		
	②Middle to high school	34	22.2 (4.7)		
	③College and above	27	24.1 (3.4)		
Marital status				1.13	0.282
	Married	92	23.0 (4.0)		
	Single	9	21.6 (3.4)		
Working status				−4.07	<0.001
	Unemployed	78	22.2 (4.1)		
	Employed	23	25.0 (2.5)		
Living arrangement				−2.13	0.068
	Living with family or friends	95	22.7(4.1)		
	Living alone	6	24.8 (2.1)		
Cancer histological type				0.52	0.597
	①Adenocarcinoma	74	23.0 (4.1)		
	②GIST	8	22.6 (2.4)		
	③Singent-cell carcinoma	19	22.0 (4.2)		
TNM cancer stage				5.39	0.006
	① 0~I	35	24.5 (2.9)	(①>③; ①>②)	
	②II	26	21.7 (5.3)		
	③III	40	22.1 (3.4)		
Gastrectomy				3.68	0.029
	①Subtotal B-I	16	23.9 (2.8)	(①>②;①>③)	
	②Subtotal B-II	65	23.2 (3.9)		
	③Total	20	20.8 (4.5)		
Adjuvant therapies				1.186	0.319
	No	50	23.3 (4.1)		
	Oral chemotherapy	27	22.7 (3.8)		
	Intravenous chemotherapy	18	21.3 (4.3)		
	CCRT	6	23.8 (2.3)		

GIST, gastrointestinal stromal tumors; CCRT, adjuvant concurrent chemoradiation therapy. * *p* < 0.05.

**Table 4:** Correlations among study variables (N = 101).

Variables	Age	Disease Duration	Symptom Severity	Depression Severity	DPD	Nutritional Status
Age	1					
Disease duration	0.01	1				
Symptom severity	−0.15	0.05	1			
Depression severity	−0.04	0.05	0.66 **	1		
Diet preparation difficulties (DPD)	−0.20 *	0.00	0.42 **	0.41 **	1	
Nutritional status	0.08	−0.03	−0.54 **	−0.40 **	−0.43 **	1

* *p* < 0.05, ** *p* < 0.01.

**Table 5:** Influencing factors of nutritional status in gastric cancer patients after gastrectomy.

Variables	*β*	*t*	*p*	Adjust *R*^2^	*F*	VIF
Regression model				0.37	20.3	
Working status	0.22	2.4 *	0.008 *			1.03
Symptom seveity	−0.42	−4.7 ***	<0.001 ***			1.22
Diet preparation difficulties	−0.22	−2.5 *	0.014 *			1.23

* *p* < 0.05, *** *p* < 0.001.

## Data Availability

Data are available from the corresponding author on reasonable request.

## Appendix A

**Table A1:** Participants’ diet preparation difficulties (N = 101).

Items	Mean	SD	Range
Fear of feeling uncomfortable after eating	2.20	1.13	1–5
Don’t know what food choices are better for my health	2.18	1.20	1–5
Don’t know how much to eat to get enough nutrition	2.18	1.18	1–5
Don’t know which foods I shouldn’t eat	2.08	1.18	1–5
Not knowing which foods to prepare will make me more appetite	1.92	1.21	1–5
Unable to decide what to prepare because of too many dietary restrictions	1.80	1.01	1–5
Sad to see myself not eating well	1.64	1.05	1–5
Can’t eat because of bloating	1.63	0.98	1–5
Worried about eating too much nutrition can make tumors bigger and more serious	1.58	0.96	1–5
Too weak to prepare food to eat	1.43	0.93	1–5
In a bad mood and do not want to eat	1.34	0.77	1–4
Having oral problems causing difficulty in eating	1.19	0.48	1–4
Some foods cannot be eaten because of Chinese medicine	1.13	0.39	1–3
Total score	22.3	8.1	13–45
